# Differential expression profile and in-silico functional analysis of long noncoding RNA and mRNA in duck embryo fibroblasts infected with duck plague virus

**DOI:** 10.1186/s12864-022-08739-7

**Published:** 2022-07-14

**Authors:** Ziyu Wu, Yue Zeng, Anchun Cheng, Anyang Sun, Mingshu Wang, Shun Chen, Mafeng Liu, Dekang Zhu, Xinxin Zhao, Ying Wu, Qiao Yang, Shaqiu Zhang, Juan Huang, Xumin Ou, Qun Gao, Sai Mao, Di Sun, Bin Tian, Ling Zhang, Zhongqiong Yin, Renyong Jia

**Affiliations:** 1 Research Center of Avian Disease, College of Veterinary Medicine, Sichuan Agricultural University, Sichuan Province, Wenjiang District, Chengdu, 611130 China; 2 Institute of Preventive Veterinary Medicine, Sichuan Agricultural University, Sichuan Province, Wenjiang District, Chengdu, 611130 China; 3 Key Laboratory of Animal Disease and Human Health of Sichuan Province, Sichuan Province, Wenjiang District, Chengdu, 611130 China

**Keywords:** Duck plague virus, LncRNA, Functional analysis

## Abstract

**Background:**

Duck plague virus (DPV), belonging to herpesviruses, is a linear double-stranded DNA virus. There are many reports about the outbreak of the duck plague in a variety of countries, which caused huge economic losses. Recently, increasing reports revealed that multiple long non-coding RNAs (lncRNAs) can possess great potential in the regulation of host antiviral immune response. Furthermore, it remains to be determined which specific molecular mechanisms are responsible for the DPV-host interaction in host immunity. Here, lncRNAs and mRNAs in DPV infected duck embryonic fibroblast (DEF) cells were identified by high-throughput RNA-sequencing (RNA-seq). And we predicted target genes of differentially expressed genes (DEGs) and formed a complex regulatory network depending on in-silico analysis and prediction.

**Result:**

RNA-seq analysis results showed that 2921 lncRNAs were found at 30 h post-infection (hpi). In our study, 218 DE lncRNAs and 2840 DE mRNAs were obtained in DEF after DPV infection. Among these DEGs and target genes, some have been authenticated as immune-related molecules, such as a Macrophage mannose receptor (MR), Anas platyrhynchos toll-like receptor 2 (TLR2), leukocyte differentiation antigen, interleukin family, and their related regulatory factors. Furthermore, according to the Kyoto Encyclopedia of Genes and Genomes (KEGG) and Gene Ontology (GO) enrichment analysis, we found that the target genes may have important effects on biological development, biosynthesis, signal transduction, cell biological regulation, and cell process. Also, we obtained, the potential targeting relationship existing in DEF cells between host lncRNAs and DPV-encoded miRNAs by software.

**Conclusions:**

This study revealed not only expression changes, but also the possible biological regulatory relationship of lncRNAs and mRNAs in DPV infected DEF cells. Together, these data and analyses provide additional insight into the role of lncRNAs and mRNAs in the host's immune response to DPV infection.

**Supplementary Information:**

The online version contains supplementary material available at 10.1186/s12864-022-08739-7.

## Background

Duck plague (DP) is caused by duck herpesvirus type I [1]. The causative agent of the disease belongs to the species Anatid *herpesvirus* I, genus *Mardivirus*, subfamily *Alphaherpesvirinae*, family *Herpesviridae* [2]. As waterfowl migrate, the disease can spread widely inside and between continents around the world, causing huge economic losses [3–6]. After DPV infection, the main injured organs in the disease seem to be the brain, liver, kidney, and lung, and the main pathological features are bleeding, inflammation, and necrosis of tissues, and organs [1, 7]. In China, the duck plague was first reported in 1959 and verified in many regions of China. And now the duck plague has not been wholly conquered in China, and there are still reports once in a while [8].

The definition of Long non-coding RNAs (lncRNAs) is non-protein-coding transcripts with more than 200 nucleotides, mainly classified into antisense lncRNAs, sense RNAs, bidirectional lncRNAs, large intergenic non-coding RNAs (lincRNAs), and intronic transcript [9, 10]. It has been found that lncRNAs have a variety of functions, including epigenetic regulation, transcriptional regulation, and post-transcriptional regulation, and unexpectedly, some transcripts annotated as lncRNAs can encode small proteins [10–12]. In addition, with the deepening of research, more researchers have started to pay more attention to that lncRNAs can also participate in the interaction between virus and host. The lnczc3h7a existed in mouse macrophage RAW264.7, can be used as a scaffold to stabilize retinoid acid-inducible gene I (RIG-I) -Tripartite Motif Containing 25 (TRIM25) complex and promote TRIM25-mediated ubiquitination of RIG-I, finally to participate in type I interferon (IFN) signal transduction and regulate RNA virus replication both in vivo and in vitro [13]. Similarly, MALAT1 can bind to TAR DNA-binding protein 43 (TDP43) in resting macrophages and inhibit IFNs expression induced by interferon regulatory Factor 3 (IRF3) [14]. In addition, there is a unique mechanism by which the virus absorbs host lncRNA to assist virus replication. After hepatitis C virus (HCV) infection, lncRNA EGOT was found to be significantly up-regulated. And Previous studies showed that EGOT had a highly significant negative correlation with innate immune-related genes that may block the antiviral response to promote virus replication [15].

Currently, there are increasing studies on lncRNAs related to avian diseases, such as the H5N1 influenza virus [16], duck enteritis salmonella [17], and duck Tembusu virus (DTMUV) [18]. However, there is a lack of reports on host lncRNAs expression induced by DPV infection. To solve this problem, the transcriptome of DPV infected duck embryonic fibroblasts (DEF) was sequenced by RNA-seq. Here we identified the differentially expressed (DE) lncRNAs and mRNAs compared with the infected and uninfected DEF cells, and predicted the target genes of DE lncRNAs using bioinformatics analysis. Based on the enrichment analysis of Gene Ontology (GO) and Kyoto Encyclopedia of Genes and Genomes (KEGG), we identified that differentially expressed genes (DEGs) in DEF cells were significantly associated with the biological processes and signal pathways.

## Results

### Characterization of DPV infection in DEFs

**Fig. 1:**
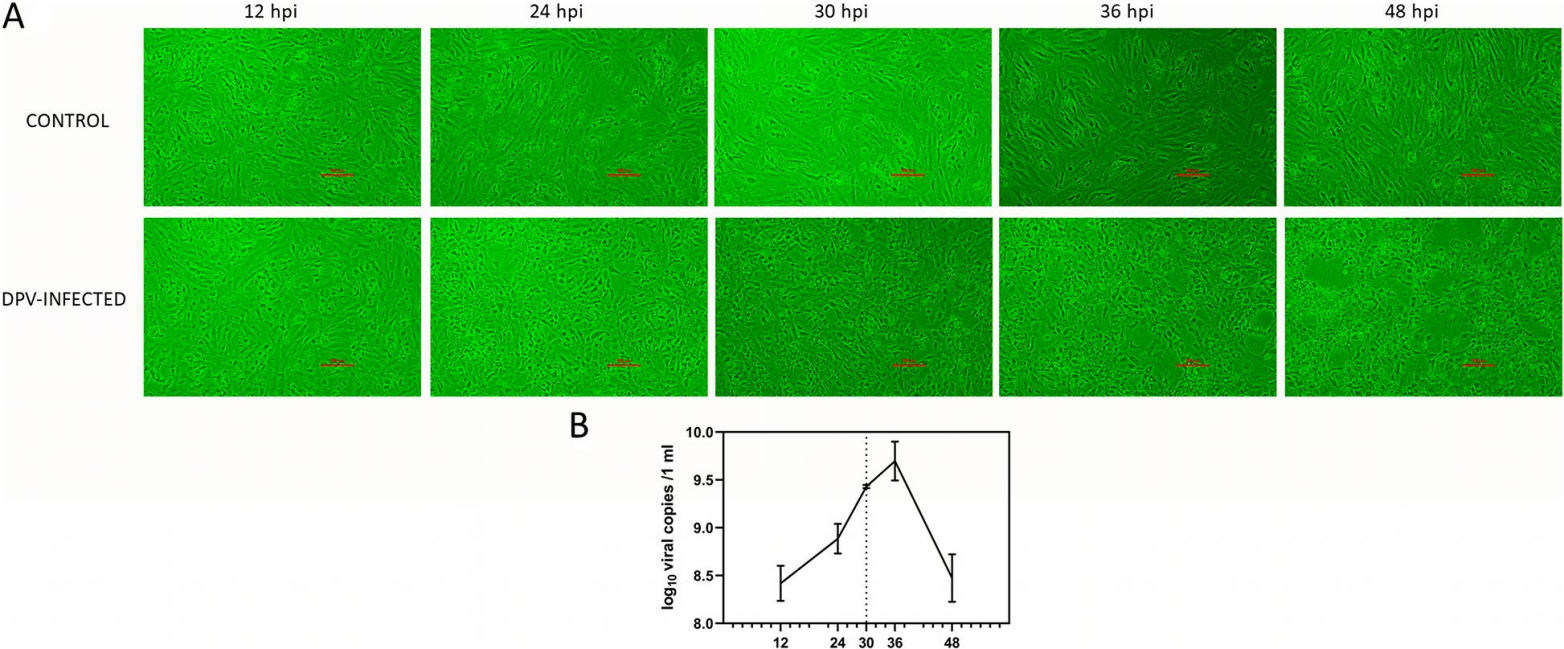
DPV infection in DEFs. **a** cytopathic effect on duck embryo fibroblast induced by DPV virus; **b** detection of DPV propagation in DEF cell culture at different time post infection by real-time PCR

At a multiplicity of infection (MOI) of 1.0, duck embryonic fibroblasts (DEF) cells were infected with DPV. In our study, CPE was observed in DPV-infected DEFs after 24 hpi, and DPV propagation in DEF cell culture began to slow down at 30 hpi (Fig. 1a, b), similar to previous studies [19, 20]. As a result, 30 hpi and an MOI of 1.0 were chosen as the condition for preparing cell samples for future high-throughput RNA-sequencing (RNA-seq).

### LncRNAs screening

After a series of data sorting and filtering processes, the number of high-quality reads per each sample is about 66,715,946–77,362,586 (Additional file 1: Table S1 and Additional file 2: Table S2). The filtered data were aligned to the reference genome. The percentage of the total aligned reads in the control group was more than 90%, while in the infection group it was more than 70% (Additional file 3: Table S3). We screened lncRNAs in the assembled transcript set [10, 21, 22]: (1) Screening the transcripts with length >  = 200 bp and exon number >  = 2; (2) Screening the transcripts with Class x/u/i (Anti-sense lncRNA/Intergenic lncRNA/Intronic lncRNA); (3) Screening for transcripts with coverage > 3 in at least one sample. That is, it appeared at least three times in one sample’s transcripts.

**Fig. 2:**
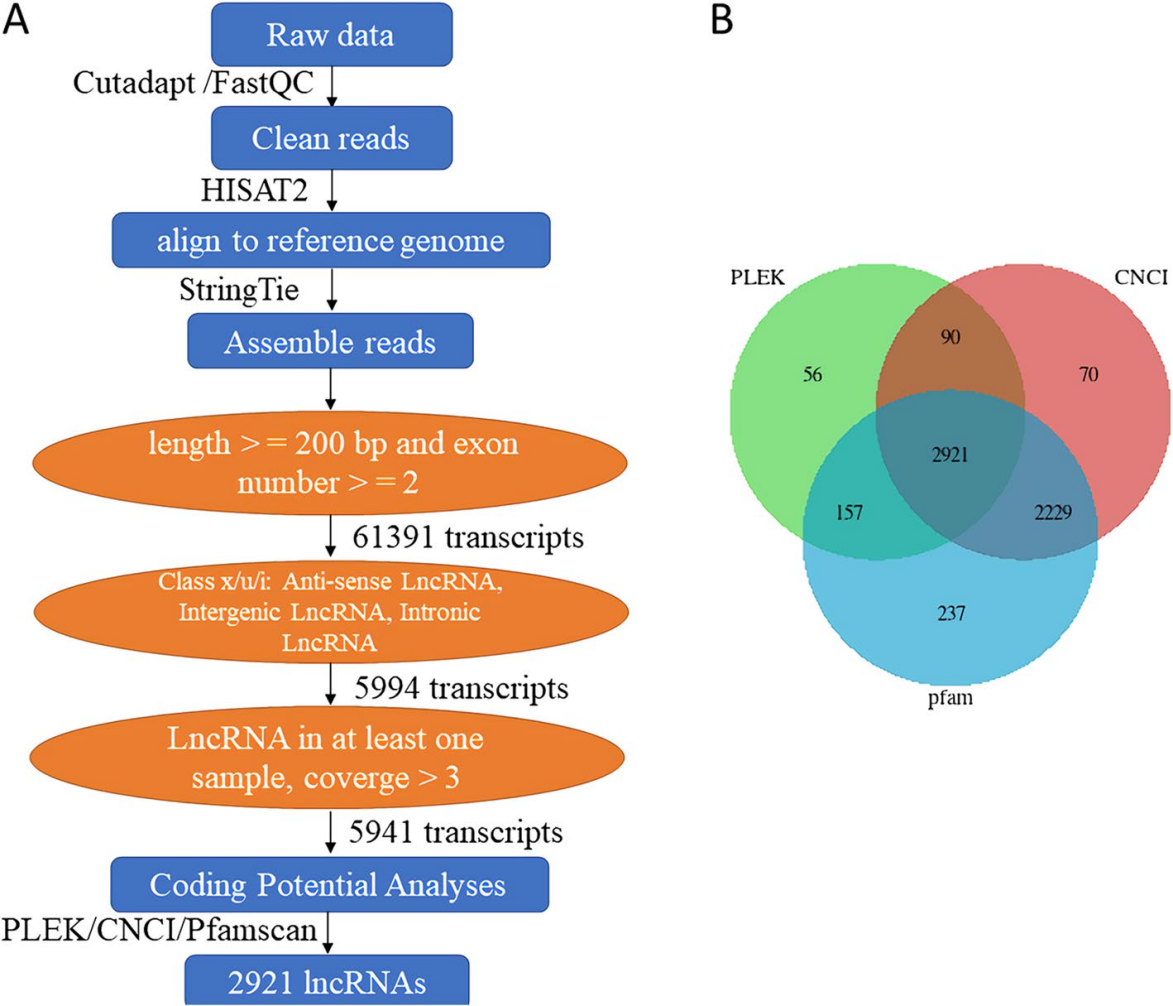
Process for identification of duck lncRNAs. **a** schematic diagram of duck lncRNAs identification; **b** the Venn diagrams showing the transcripts with non-coding potential predicted by plek, CNCI, and Pfam

A total of 61,391 long transcripts were screened out in the first step, 5994 long transcripts were screened out in the second step, and finally, 5941 long transcripts were screened out for follow-up analysis (Fig. 2a). Further analysis of the coding potential of the candidate lncRNAs was performed to get high-credibility lncRNAs. A total of 2921 novel lncRNAs with high-reliability were found by the following three methods: plek, CNCI, and pfamscan (Fig. 2a, b).

### Comparative analysis of lncRNAs and mRNAs

**Fig. 3:**
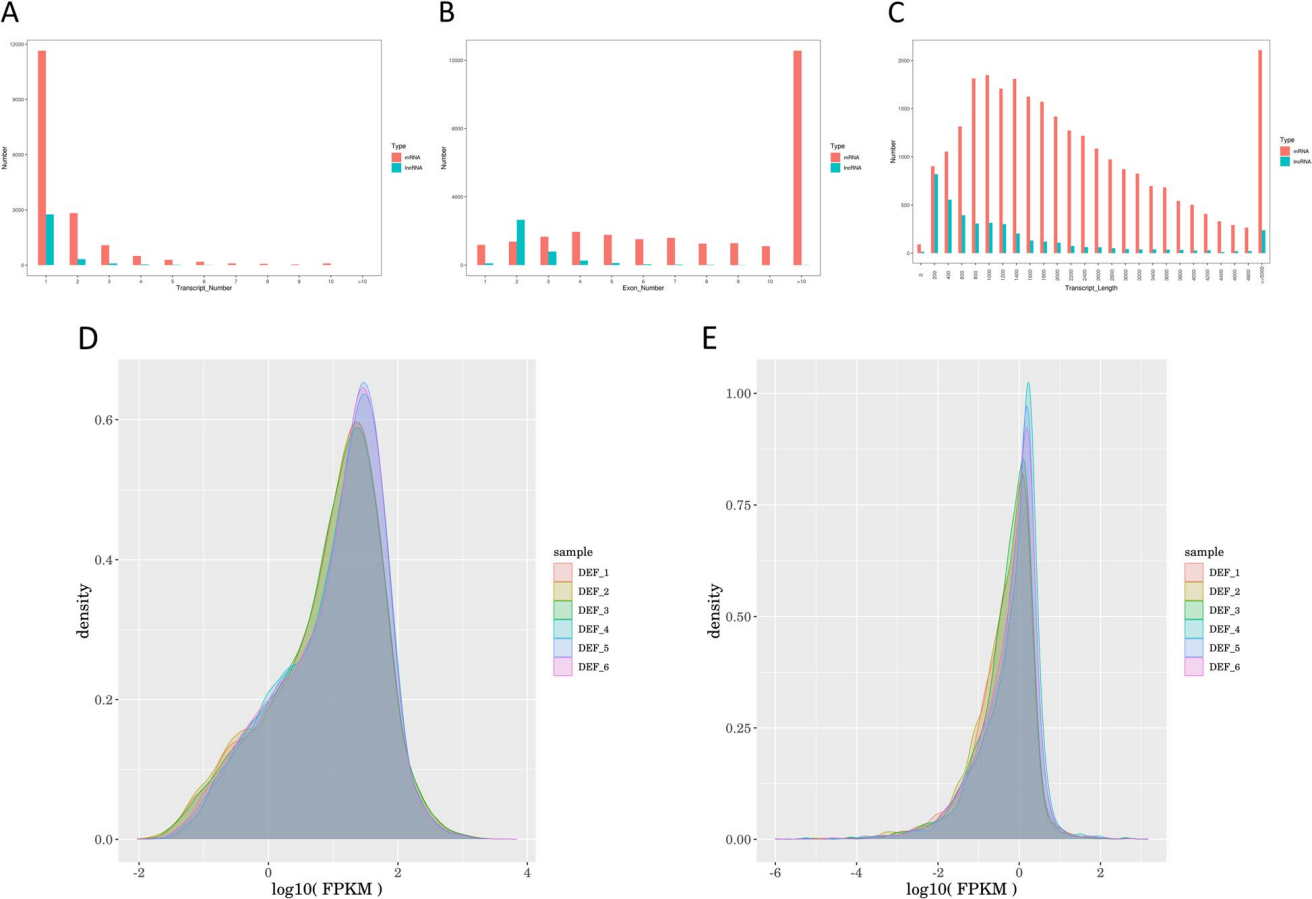
Comparative analysis of lncRNAs and mRNAs. **a** number comparison between lncRNAs and mRNAs transcripts; **b** length comparison between lncRNAs and mRNAs; **c** comparison of the exon number of lncRNAs and mRNAs; **d** FPKM density distribution of mRNA; **e** FPKM density distribution of lncRNA

The structures of known and unknown lncRNAs and mRNAs were compared. On the one hand, the result showed the molecular differences between lncRNAs and mRNAs, on the other hand, it verified whether the predicted lncRNAs accord with the general characteristics. As shown in Fig. 2, the results showed that on the whole, the number of mRNA transcripts was widely distributed, ranging from 1 to 10, mainly in the range of 1–2, whereas the number of lncRNAs transcripts was only in the range of 1–6, and mainly a single transcript (Fig. 3a). In terms of length analysis, the major length of mRNAs was in the range of >  = 5000 and 800–1600, while lncRNAs were chiefly distributed in the range of 200–300 and showed a stepwise downward trend in the range of 400–4800, but there was a certain quantitative trend rising in the range of >  = 5000 (Fig. 3b). Most mRNAs contained a large number of exons, mainly in the range of > 10, while the number of exons in lncRNAs was small, only 2–3 (Fig. 3c). Based on the results of FPKM (fragments per kilobases per million fragments) density distribution, we observed that the FPKM distribution of mRNAs was gathered at 1–2, while the expression level of lncRNA is lower (Fig. 3d, e).

### Deep sequencing results

**Fig. 4:**
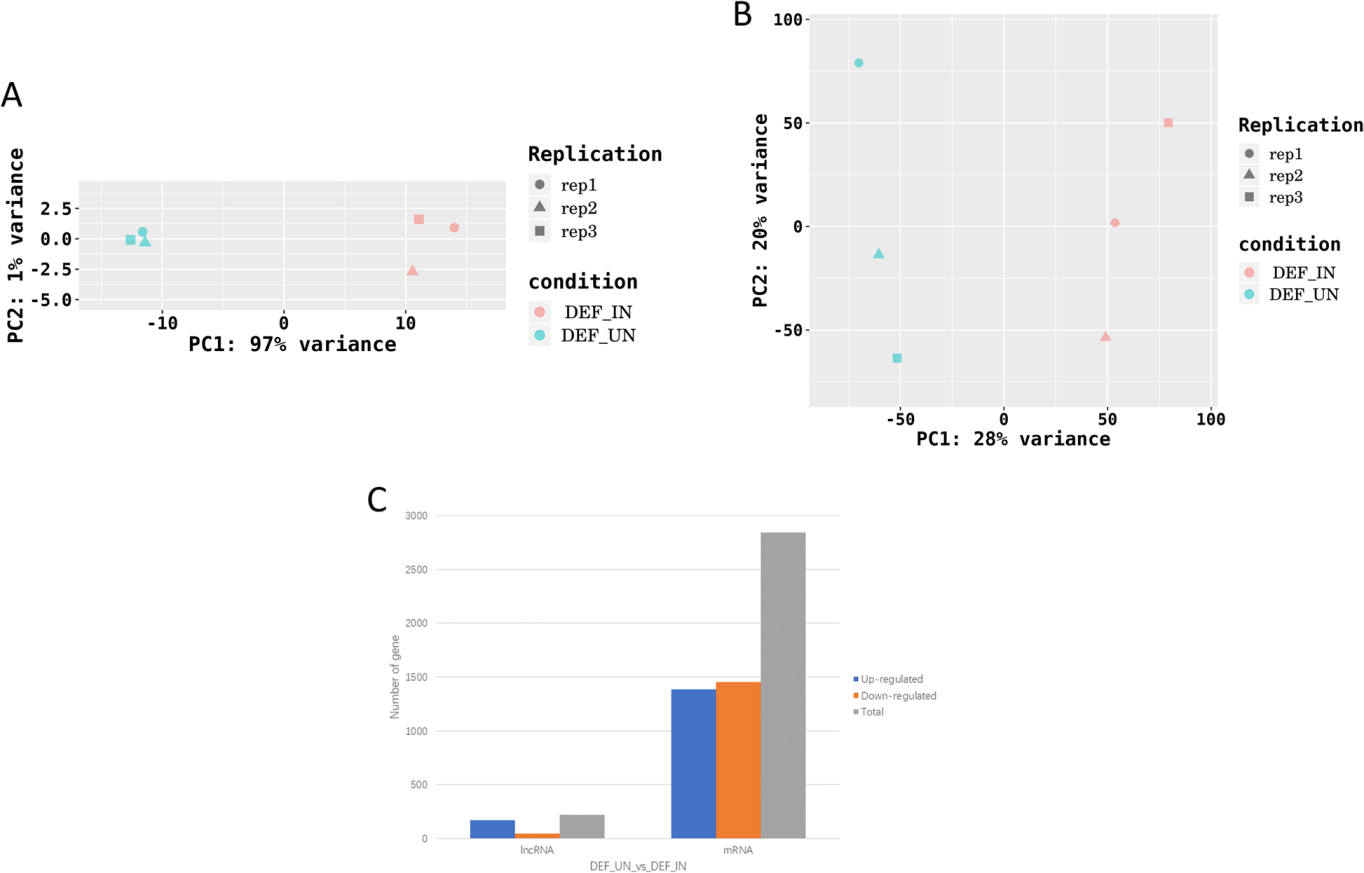
Results of correlation test between samples. **a** PCA results of mRNAs, in the chart, The abscissa is the first principal component and the ordinate is the second principal component. Different shapes in the figure represent different samples, and different colors represent different groups; **b** PCA results of lncRNAs; **c** the histogram summarizes the distribution of up-regulated and down-regulated mRNA and lncRNA

We also performed PCA (Principal Component Analysis) to check the correlation between samples. And it was found that there were greater differences in the total lncRNAs of biological repeat samples in each treatment group, compared with mRNAs (Fig. 4a, b).

**Fig. 5:**
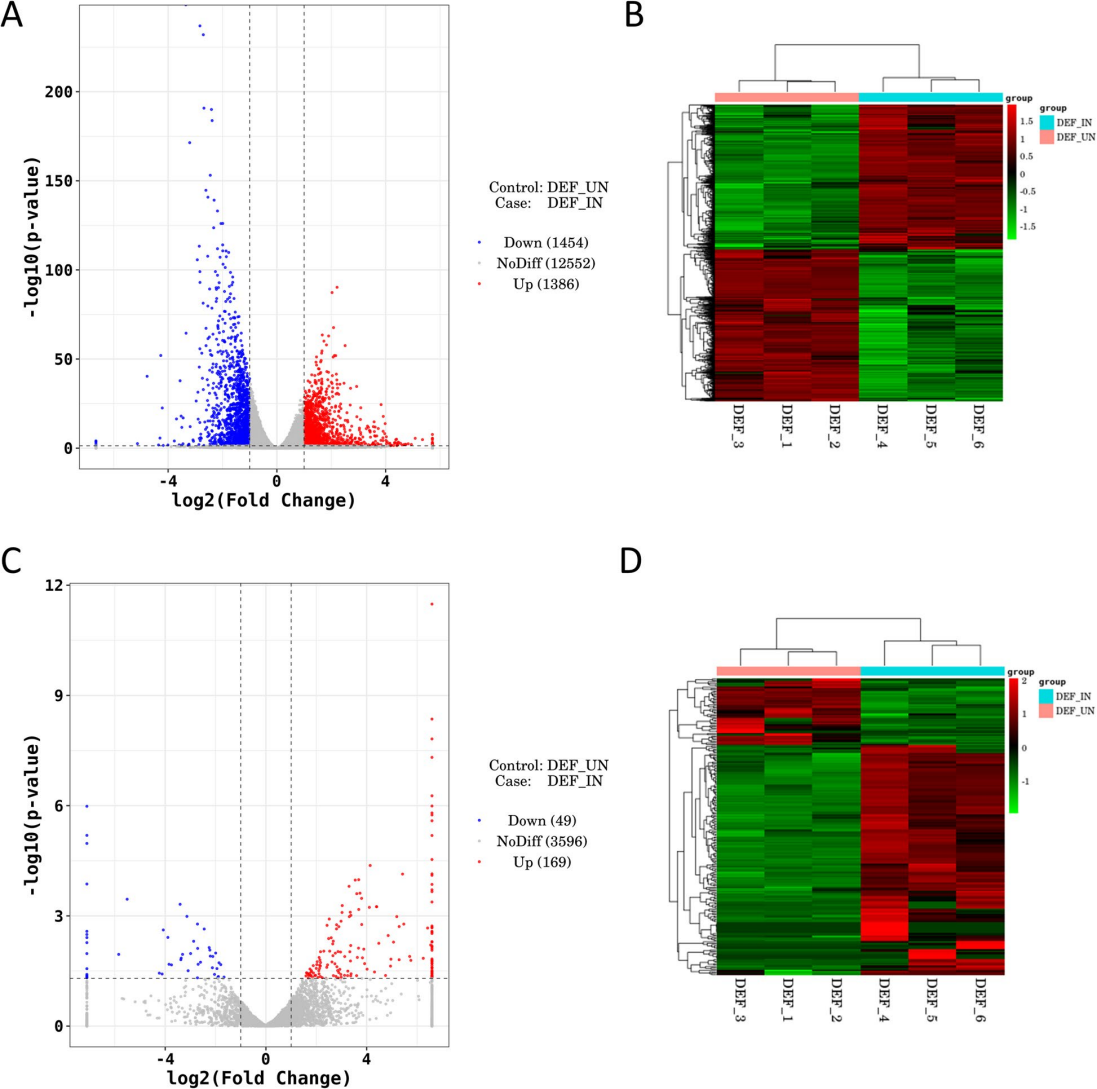
Differential analysis of lncRNA/mRNA expression in DEFs infected with DPV. **a** The volcano plot of the DE mRNAs in DEFs infected with DPV; **b** the heat map of the DE mRNAs in DEFs infected with DPV; **c** the volcano plot of the DE lncRNAs in DEFs infected with DPV; **d** the heat map of the DE lncRNAs in DEFs infected with DPV

The calculation method of *P* value of DESeq2 is very strict. Therefore, we choose |log2 fold change (infected/mock in expression) |> 1 and *p*-value < 0.05 as the screening standard, hoping to get more DEGs for analysis. The differential expression analysis results indicated that compared with the control group (Fig. 4c), we identified 2840 DE mRNAs in DEF after DPV infection, of which 1386 were up-regulated and 1454 were down-regulated (Additional file 4: Table S4), and 218 DE lncRNAs, of which 169 were up-regulated and 49 were down-regulated (Additional file 5: Table S5 and Additional file 6: Table S6). Besides, we used volcano plots and heat maps to show DE lncRNAs and DE mRNAs (Fig. 5a-d).

Among them, the mRNA with the most significant difference in expression level are actin and myosin-related factors. Further, some DE mRNAs have been identified as immune-related molecules, which can be classified into the following three categories: pattern recognition receptors (PRRs) (such as Macrophage mannose receptor 1 and Anas platyrhynchos toll-like receptor 2), leukocyte differentiation antigens (like CD81, CD82, CD36, and CD2 associated protein) and immune cell-derived cytokines and their related regulatory factors (such as interleukin 15, interleukin 21 receptor, interleukin 6 family cytokine, interleukin 23 receptor, interleukin-9 receptor-like gene, interleukin 18 receptor accessory protein, interleukin 19, interleukin 6 signal transducer, cytokine-inducible SH2 containing protein, and Cytokine-like 1 gene). Interestingly, most of these genes were up-regulated in the infection group (Additional file 4: Table S4).

### Potential interacting genes prediction

**Fig. 6:**
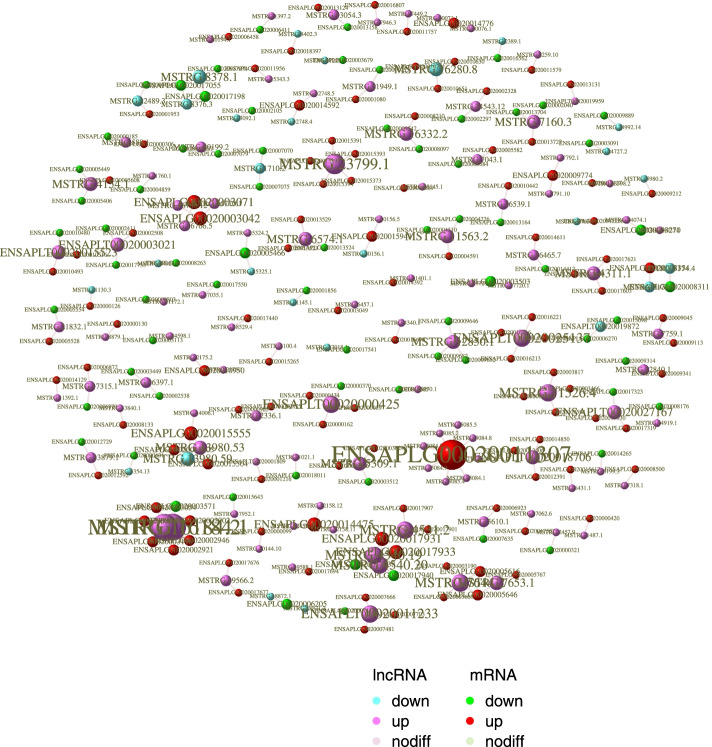
Prediction of potential cis-target genes of lncRNAs. In the chart, we use blue to represent lncRNA, red to represent mRNA, and wiring to represent target relationship by the Igraph package (https://igraph.org/)

**Fig. 7:**
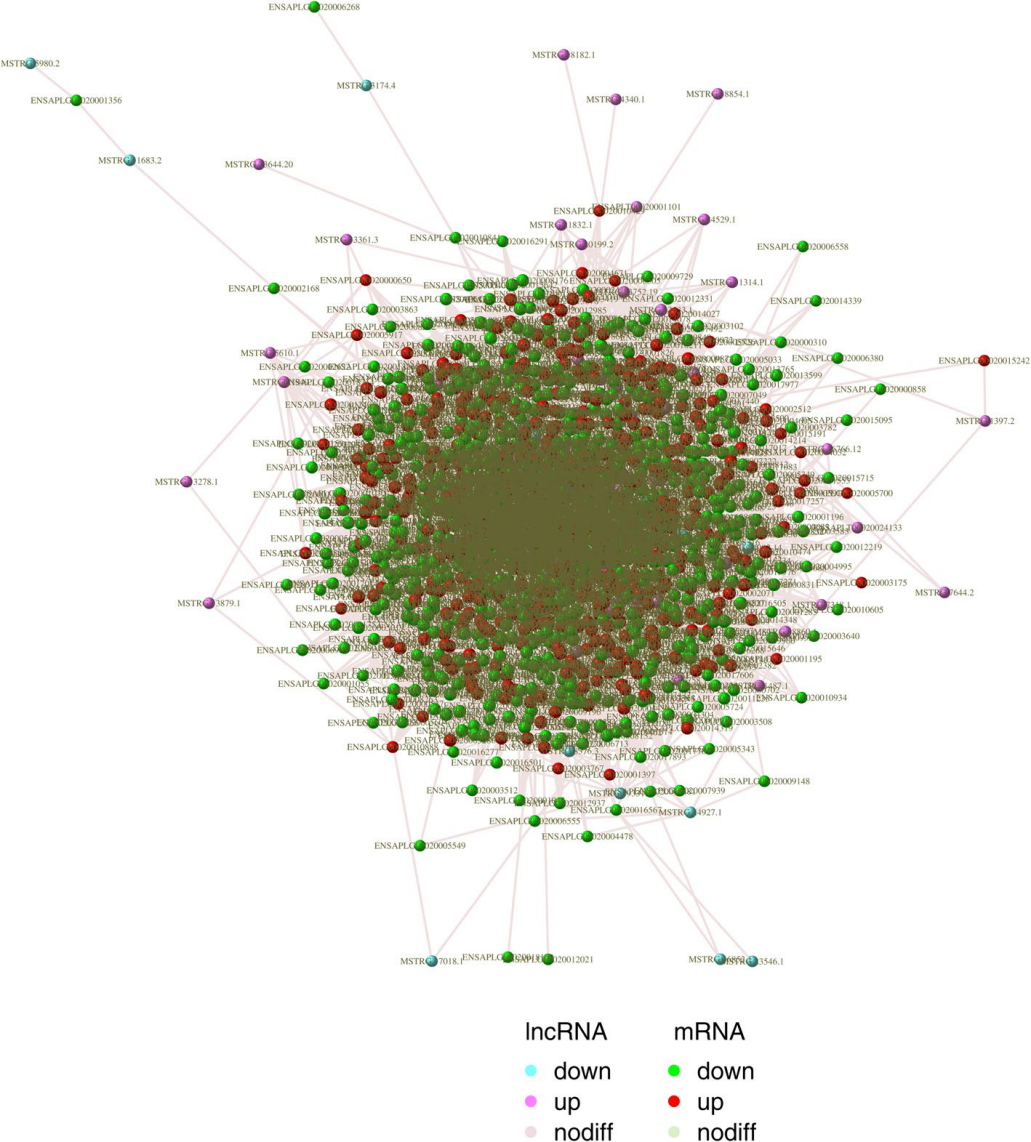
Prediction of potential trans-target genes of lncRNAs. In the chart, we use blue to represent lncRNA, red to represent mRNA, and wiring to represent target relationship by the Igraph package (https://igraph.org/)

Then, we predicted potential interacting genes of lncRNA in cis- and trans-regulation, as shown in figures (Fig. 6 and Fig. 7). The previous study has shown that lncRNA can regulate the expression of adjacent genes by recruiting regulatory factors and competing for TF (Transcription Factor) and Pol ll (RNA polymerase II) required for transcription. Or the transcription of lncRNA can interfere with the cis-regulatory element of mRNA, contained in the lncRNA encoded region [10]. Besides, lncRNAs can also regulate chromatin state and structure, organization of nuclear, and trans-acting factors to regulate distal gene expression [10]. And it was found that in the predicted results, 3762 lncRNAs (of a total 3814) were predicted to have cis regulatory function, and1388 lncRNAs could act in trans-regulation. Interestingly, 1262 lncRNAs have a large number of possible associated genes, whether in cis-regulation or trans-regulation (Additional file 7: Table S7 and Additional file 8: Table S8).

### Functional enrichment analysis of DE lncRNAs

**Fig. 8:**
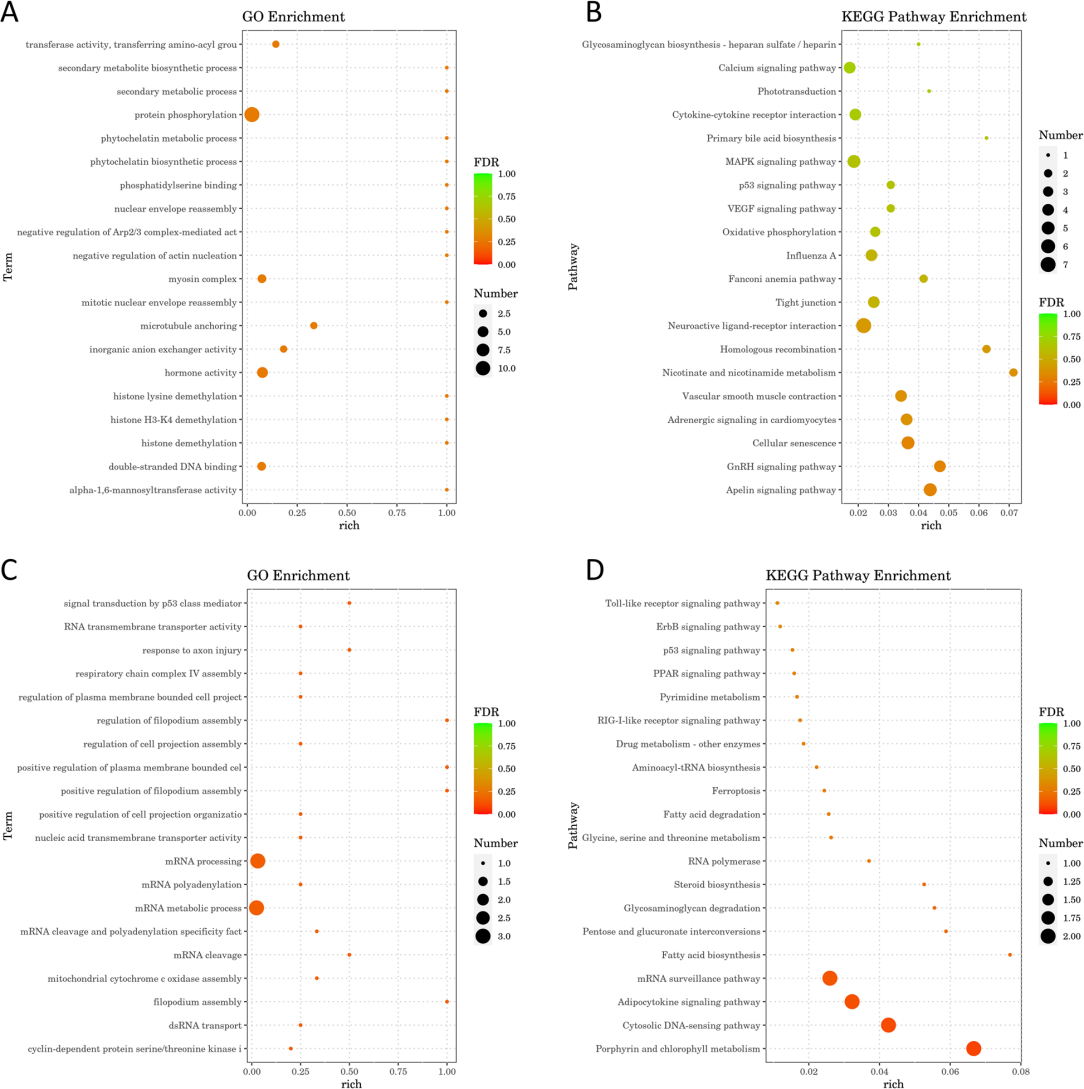
Functional enrichment analysis for predicted target genes of DE lncRNAs. **a** GO enrichment analysis bubble chart: display the top 20 GO terms with the most significant enrichment in predicted cis-acting pair; **b** KEGG enrichment analysis bubble chart: display the top 20 KEGG pathways with the most significant enrichment in predicted cis-acting pair; **c** GO enrichment analysis bubble chart: display the top 20 GO terms with the most significant enrichment in predicted trans-acting pair; **d** KEGG enrichment analysis bubble chart: display the top 20 KEGG pathways with the most significant enrichment in predicted trans-acting pair

The GO and KEGG analyses were conducted to predict the biological regulatory function of DE duck lncRNAs (Fig. 8a-d). Based on the GO enrichment analysis, we found cis-associated genes were significantly referred to the cell biological items (*p* < 0.05) such as hormone activity, double-stranded DNA binding, protein phosphorylation, microtubule anchoring, and transferase activity, while trans-acting potential target genes were significantly enriched in (*p* < 0.05) RNA process, filopodium and cell cycle, involving cell biological regulation, signal transduction, biosynthesis, response to external stimulus, and cell process (Additional file 9: Table S9 and Additional file 11: Table S11). Besides, there are some GO terms that may be associated with the process of virus infection, such as Wnt signaling pathway (GO:0,030,111, *p* < 0.05; GO:0,030,177, *p* < 0.05; GO:0,016,055) in trans-regulation, and IL-2 terms (GO: 0,004,911, *p* < 0.05; GO: 0,019,976, *p* < 0.05), JAK/STAT signaling pathway (GO: 0,007,259, GO: 0,097,696) and Wnt signaling pathway (GO:0,030,111, GO:0,030,178) in cis-regulation.

At the same time, KEGG analysis showed that multiple signal pathways associated with inflammation and host antiviral immune response were enriched in (Additional file 10: Table S10 and Additional file 12: Table S12), for instance, Toll-like receptor signaling pathway, C-type lectin receptor signaling pathway, RIG-I-like receptor signaling pathway, and NOD-like receptor signaling pathway were found in both trans- and cis-regulation. Also, the mitogen-activated protein kinase (MAPK) signaling pathway and Cytokine-cytokine receptor interaction were enriched at cis-regulation.

### Functional enrichment results of DE mRNAs

**Fig. 9:**
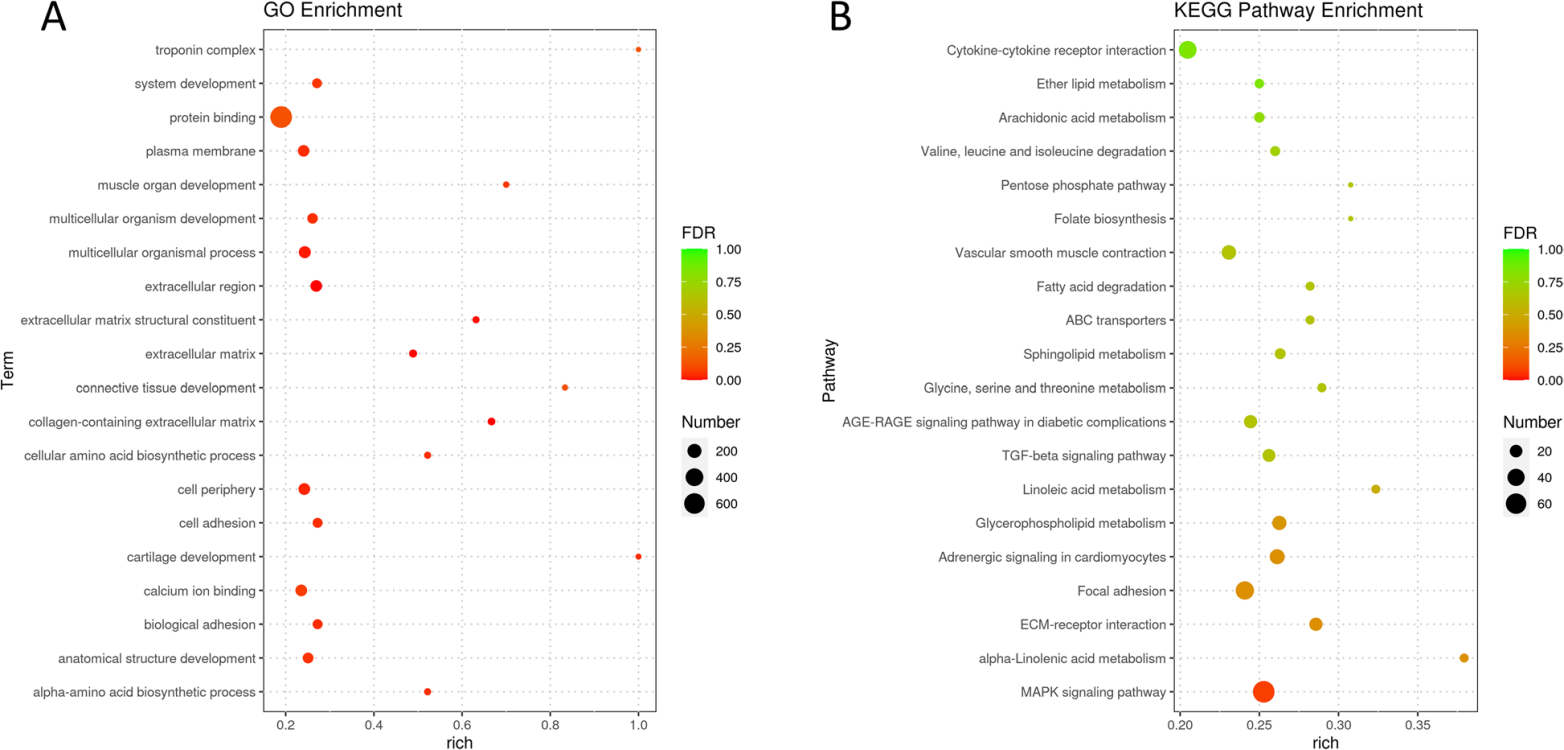
Functional enrichment analysis for target genes of DE mRNAs. a, GO enrichment analysis bubble chart: display the top 20 GO terms with the most significant enrichment; b, KEGG enrichment analysis bubble chart: display the top 20 KEGG pathways with the most significant enrichment

In addition, we performed functional analyses of DE mRNAs (Fig. 9a, b). As shown in the results, DE mRNA was significantly related to (*p* < 0.05) biological development and biosynthesis (Additional file 13: Table S13). There are also a large number of enriched GO terms related to immunity, such as regulation of defense response (GO:0,031,347, *p* < 0.05), response to virus (GO:0,009,615, *p* < 0.05), defense response to virus (GO:0,051,607, *p* < 0.05), and negative regulation of I-kappaB kinase/NF-kappaB signaling (GO:0,043,124, *p* < 0.05).

The results of KEGG pathway analyses revealed that the enrichment results were significantly related to (*p* < 0.05) the signal transduction, metabolism, and biomembrane (Additional file 14: Table S14). And the top enriched KEGG pathway was MAPK signaling pathway (*p* < 0.05). Similar to the enrichment results of lncRNA predicted target genes, Cytokine-cytokine receptor interaction, Wnt signaling pathway, NOD-like receptor signaling pathway, Toll-like receptor signaling pathway, and RIG-I-like receptor signaling pathway were all present in the KEGG pathway analyses.

### Establishment of predicted competitive endogenous RNA (ceRNA) network

LncRNA-miRNA plays a significant role in gene expression regulation. LncRNA with microRNA response elements (MREs) can be used as a competing endogenous RNA (ceRNA) to inactivate miRNA by binding to miRNA [23]. Because most lncRNAs are structurally similar to mRNAs, miRNA may also negatively regulate the expression of lncRNA through a similar mechanism of action on mRNA, and then plays a series of biological roles [24]. At the same time, miRNA can also promote the expression of specific lncRNA [25]. In this study, we obtained the prediction results of the interaction between DE lncRNAs and miRNAs encoded by DPV virus [26]. And we found that each miRNA was predicted to interact with a large number of lncRNAs, and many lncRNAs could also be targeted by multiple miRNAs at the same time (Additional file 15: Table S15 and Additional file 16: Figure S1), for instance, 24 viral miRNAs were all predicted to target ENSAPLT00020019865.

### Validation of DE lncRNAs and mRNAs by qRT-PCR

To validate the DEGs in RNA-seq results, we randomly selected 12 DEGs (6 lncRNAs and 6 mRNAs) for qRT-PCR analysis. And it was found that among the 6 lncRNAs, there were 5 up-regulated lncRNAs and 1 down-regulated lncRNA (MSTRG.24727.2) by qRT-PCR analysis, differential expression patterns of which were consistent with RNA-seq results (Additional file 17: Figure S2). Similarly, the expression trend of the chosen mRNAs was in accordance with the results of RNA-seq analysis (Additional file 18: Figure S3).

## Discussion

With an in-depth understanding of lncRNA, it has been found that virus infection can affect the expression of host lncRNAs, and lncRNAs can also play a unique role in the process of virus infection. For example, RIG-I-dependent IAV upregulated noncoding RNA (RDUR), induced by Influenza A virus (IAV) infection, can positively regulate the signal transduction of type I interferon (IFN-1) by affecting IRF3 and further upregulates the expression of several vital antiviral molecules. It is concluded that RDUR is a critical regulator of innate immunity against viral infection [27]. And lncRNA-GM can bind to glutathione S-transferase M1 (GSTM1), block the interaction between GSTM1 and TANK binding kinase 1 (TBK1), and reduce GSTM1-mediated glutathione. Finally, the deficiency of lncRNA-GM in mice increases sensitivity to viral infection and interferes with the production of IFN-I [28]. There are also lncRNA-ACOD1, which are effectively induced by several viruses. This lncRNA can promote viral replication by activating the glutamic acid transaminase 2 (GOT2), a metabolic enzyme. Interestingly, the enhancement of GOT2 activity by lncRNA-ACOD1 is necessary for virus replication [29]. DPV is a pathogen causing a hot, acute, and septic infectious disease in ducks, geese, and swans, with a great number of economic losses [1]. Moreover, the regulatory mechanism between DPV and host remains to be discussed. Here we analyzed the transcriptome of DPV infected DEF and obtained 218 DE lncRNAs and 2840 DE mRNAs at 30 hpi.

In our study, we found that some DE mRNAs were closely related to immune regulation, such as toll-like receptor 2 (TLR2), mannose receptor (MR) family, T cell surface antigen, interleukin family, and their related regulatory factors. There are many reports showed that the above factors are involved in the regulation of the host antiviral immune response. For example, the coordinated activation of TLR2 and TLR9 in trigeminal ganglion exists in the defence response of mice to the herpes simplex virus-1 (HSV-1), while the expression of TLRs in HSV-1 infected mice is significantly increased [30]. And it has been reported that innate immune recognition of vaccinia virus (VV) is mediated by TLR2 [31]. However, it is also reported that the above immune factors can participate in the interaction between the virus and the host. For example, macrophage mannose receptor (MMR) can participate in the infection of mouse macrophages by influenza virus, which is necessary for endocytic uptake of virus [32]. Similarly, CD81 can promote the entry of the HCV into host cells [33], and CD2 related protein (CD2AP) can promote the assembly of HCV [34]. Meanwhile, the differential expression level of actin-related factors changed significantly. Actin is abundant in eukaryotic cells and previous studies have shown that actin could intimately interact with lentiviruses [35]. Also, cofilin, one of the actin-binding proteins, was found to be involved in the process of HSV infection [36]. The findings suggested that DE mRNAs may similarly regulate the pathogenicity of DPV.

In addition, the functional analysis indicated that the target genes and DEGs significantly related to (*p* < 0.05) biological development, biosynthesis, metabolism, and signal transduction. At the same time, some targets were enriched in IL-2 terms, Wnt signaling pathway, JAK/STAT pathway terms, and other important GO terms. Interleukin-2 (IL-2) is a growth factor of various cells, which is closely related to the growth and differentiation of T cells, the development of functional B cells, and the activation of NK cells [37]. Studies have proved that duck IL-2 is an important immunomodulatory molecule, which has been fully verified in chickens and mammals [38]. Similarly, JAK/STAT pathway is an important cytokine signal transduction pathway, which is activated by a variety of cytokines, growth factors, and receptors. And it involves many biological processes such as immune regulation, inflammatory response, cell proliferation, differentiation, apoptosis, angiogenesis, oxidative stress, and tumor formation [39]. Also, it is related to the process of a variety of viruses infection, such as porcine deltacoronavirus (PDCoV) [40], hepatitis B virus (HBV) [41]. And the Wnt pathway is an important signal pathway, which participates in the regulation of a variety of cell processes, which is closely related to the development of the body, the maintenance of homeostasis, and the occurrence and development of a variety of diseases, including viral diseases [42–44]. Therefore, the above results indicated that there may be IL-2, Wnt pathway or JAK/STAT pathway-dependent manners in DEF after DPV infection to deal with virus infection. In addition, after the KEGG pathway enrichment analysis, we also found target genes were significantly enriched in MAPK signaling pathway (*p* < 0.05), RIG-I-like receptor signaling pathway, Toll-like receptor signaling pathway, and other immune-related pathways. It has been reported that MAPK pathway activation is important for the replication of many viruses, such as foot-and-mouth disease virus (FMDV) [45], bovine herpesvirus type 1 (BoHV-1) [46, 47], and KS associated herpesvirus (KSHV) [48]. Perhaps there is a similar mechanism in DPV infected DEF cells. Further, both RIG-I and TLR can participate in NF-κB signal transduction after detecting viruses to regulate host antiviral immunity [49, 50]. This is also consistent with the enrichment of NF-κB signaling pathway-related GO terms (GO:0,043,124, *p* < 0.05) in the functional analysis of DEGs.

In addition, we also analyzed the potential viral miRNAs that may interact with DE lncRNAs based on the DPV-encoded miRNA sequence spectrum of Wu et al. [26]. Previous studies have also shown that viral miRNAs can also play an important role in virus invasion and replication process [51–53], and that lncRNA could post-transcriptionally regulate target genes by microRNA response elements (MREs) [23]. Here, we would like to further understand whether DE lncRNAs may be involved in a similar process during DPV infection. However, these analysis results need further experimental exploration to prove.

## Conclusion

In our study, we screened out numerous DE lncRNAs and mRNAs by RNA-seq, formed a potential target interaction network in silico analysis, and predicted potential biological functions. LncRNA continues to be hot in the field of scientific research in recent years because of its various gene regulation mechanisms and a wide range of biological functions. We obtained a total of 218 DE lncRNAs and 2840 DE mRNAs, which were beneficial to understand the cellular mechanism of DPV infection with DEF.

## Methods

### Cell and virus

Primary DEF cells were obtained from 9-day-old duck embryos after trypsin digestion for subsequent experiments and cultured in Dulbecco's modified eagle medium (DMEM) supplemented with 10% fetal bovine serum (Gibco, USA) at 37 °C with 5% CO2. The DPV CHv (GenBank accession No. JQ647509) used in this study was kept in our laboratory. At a multiplicity of infection (MOI) of 1.0, duck embryonic fibroblasts (DEF) cells were infected with DPV or mock-infected with DMEM as control.

### RNA isolation and RNA-Seq

All experiments were carried out in triplicate. At 30 h post-infection (hpi) [19, 20], total cellular RNAs were extracted using the RNAiso plus reagent (TaKaRa, Japan). The purity, integrity, and concentration of total RNA samples were evaluated and tested by NanoDrop NC2000 Spectrophotometer (Thermo Fisher Scientific, USA) and Agilent RNA 6000 nano kit (Agilent Technologies, USA). Rin value (RNA integrity number) was automatically generated by Agilent 2100 Bioanalyzer (Agilent Technologies, USA) which represents the quantitative value of RNA integrity. The quality test results showed that the RIN values of the samples were 10, which met the requirements of database construction and sequencing. All the infected and uninfected samples were sequenced by Shanghai Personal Biotechnology (Shanghai, China).

### Reads filtering and mapping

We calculate the data of each sample (Raw Data) separately, including sample names, Q30, percentage of ambiguous reads, Q20 (%), and Q30 (%). Remove low-quality reads using Cutadapt software (http://cutadapt.readthedocs.io/en/stable/). Then the filtered reads were aligned to the reference genome (Anas_platyrhynchos.ASM874695v1.dna.toplevel.fa, http://asia.ensembl.org/Anas_platyrhynchos/Info/Annotation) using the HISAT2 software (http://ccb.jhu.edu/software/hisat2/index.shtml) [54]. Based on the preliminary gene mapping results, we assembled transcripts by StringTie software (http://ccb.jhu.edu/software/stringtie/) [55]. We used the following three methods for coding potential analysis: plek (https://sourceforge.net/projects/plek/files/) [56], CNCI (https:// github.com/www-bioinfo-org/CNCI) [57], pfamscan (https://www.ebi.ac.uk/seqdb/confluence/display/JDSAT/PfamScan+Help+and+Documentation) [58], and considered that the new transcripts without coding potential determined by the three software were high-reliability lncRNAs.

### Identification of differentially expressed RNAs

The correlation of gene expression level between biological repeat samples is an important index to test the reliability of experiments and the rationality of sample selection. We used DESeq Software package(http://www.bioconductor.org/packages/release/bioc/html/DESeq.html) to carry out PCA (principal component analysis) for each sample according to the expression amount [59, 60]. Then, we used HTSeq statistics (https://htseq.readthedocs.io/en/master/) to obtain the reads count on each gene as the original expression level [61]. In order to facilitate comparison of gene expression of different genes in different samples, we used FPKM (fragments per kilobases per million fragments) as the standard of measurement by the Cufflinks program (http://cole-trapnell-lab.github.io/cufflinks/) [62]. We also used Stringtie software to obtain the reads count of lncRNAs and then homogenized the expression level with FPKM. The differential expression analyses of RNAs were performed by DESeq (http://www.bioconductor.org/packages/release/bioc/html/DESeq.html) [59, 60]. The conditions for screening DEGs were as follows: |log2 fold change (infected/mock in expression) |> 1 and *p*-value < 0.05. We used the phatmap software package (https://CRAN.R-project.org/package=pheatmap) to conduct a two-way cluster analysis on the Union and samples of different genes in all comparison groups. According to the expression level of the same genes in different samples and the expression patterns of different genes in the same sample, the Euclidean method was used to calculate the distance, and the hierarchical clustering longest distance method (complete linkage) was used for clustering.

### Target genes prediction of lncRNAs

Increasing researches showed that the cis-regulatory function of lncRNAs is associated with the protein-coding genes near their genomic locations [10, 19, 63]. Therefore, to explore the regulatory function of lncRNAs, the protein-coding genes within 100 kb upstream and downstream of the lncRNA genes were searched and thought to be the cis-regulatory target genes of their corresponding lncRNAs.

In contrast, the basic principle of trans-regulatory target genes prediction holds that the function of lncRNA is dependent on its co-expressed protein-coding genes [10, 19, 63]. The conditions of correlation coefficient | correlation |> 0.95 and *p*-value < 0.05 were used to screen the trans-regulatory relationship between lncRNAs and mRNAs.

### In-silico gene functional prediction analysis

We used the TopGO (http://www.bioconductor.org/packages/release/bioc/html/RamiGO.html) for GO enrichment analysis of DEGs and target genes [64]. Based on the DEGs and target genes annotated by the GO database (http://geneontology.org/), then we calculated the p-value of DEGs and target genes by hypergeometric distribution method (p-value < 0.05 is regarded as the standard of significant enrichment), and took the whole genome as the background to screen the GO terms with significant enrichment of DEGs and target genes, to determine the main biological functions performed by DEGs and target genes [65]. Furthermore, we predicted the associated pathways of DEGs and target genes by the KEGG database (http://www.kegg.jp/) [66, 67]. Similarly, the criterion for significant enrichment is *p*-value < 0.05.

### Competitive endogenous RNAs (ceRNAs) regulatory network construction

We used miRanda software (https://www.cs.kent.ac.uk/people/staff/dat/miranda/) and the DPV-encoded miRNA sequence spectrum to predict target miRNAs for lncRNAs respectively, and finally obtained the predicted miRNA-lncRNA pairs to have a targeting relationship [24, 68]. The final lncRNA-miRNA regulatory networks were visualized with Cytoscape software (https://cytoscape.org/) [69].

### QRT -PCR Analysis

We randomly selected several DEGs and determined the expression changes by Quantitative Real-time PCR (qRT-PCR) to verify the validity of sequencing data. The extracted total RNA was used to reverse transcribed to cDNA by PrimeScript™ RT reagent Kit with gDNA Eraser (Perfect Real Time) (TaKaRa, Japan). The expression of DEGs was detected by the CFX Connect real-Time PCR Detection System (Bio-Rad, USA) and TB Green® Premix Ex Taq™ II(Tli RNaseH Plus) (TaKaRa, Japan). The β-actin gene was used to standardize the expression of DEGs, and calculate relative expression levels by the 2-△△Ct method [70]. The primers used in this study were synthesized by Huada Company (Guangdong, China) and shown in Table 1.

**Table 1:** Primer used in qRT-PCR

DEGs name	Forward (F) or Reverse (R)	Sequence (5’-3’)
MSTRG.7043.1	F	TGGCAGACACTTCAGCACTCT
	R	GGTAAATCCCTAGTTCAGCAGTCAG
MSTRG.12158.11	F	ACTGTCCTACAAGCGGCGTAA
	R	CTCGTAGTTCTCTGGCGGATACT
MSTRG.23371.5	F	GTAATCAGCCTCTCAATCACCTTCAC
	R	TGTCAACCTCCAGCAACTTCTCT
MSTRG.23546.3	F	TGATGACAGGATGAGGAGGAATGG
	R	GAGCCAATAAGTCACAGAATCACAGA
MSTRG.16184.2	F	AGGAGCCGTTGGATGGTGTAAT
	R	ATTCACTCTTCTCCACGAGGTAGG
MSTRG.24727.2	F	CTACCACAGCAATGTCTTCCACTACTC
	R	CAGCATCACAATCACTTACCACAAGGA
ENSAPLG00020017612	F	TTGATATTCGTAAGGACCTGTATGC
(ACTC1)	R	GCGGTGGACAATGGATGGA
ENSAPLG00020011270	F	TCCTCTGCTGCCTCTTCTG
(SRD5A2)	R	GGAGATCGCTGTGAATGTTGA
ENSAPLG00020002523	F	TGAAGATGACGATGAGGAGGAG
(FANCI)	R	TACACCAACACCAGAAGACAGA
ENSAPLG00020012559	F	CTTGGTGATGCTGACGATGGA
(RCC1)	R	TTGTTGCCTCTGCCTCTGAC
ENSAPLG00020016685	F	GCCAAGCCATTCCACCAGAT
(DCN)	R	TGTAAGTCCAGCAGAGTTGTATCAG
ENSAPLG00020004383	F	CTGCGACGAGATGAACGACAT
(SFRP2)	R	AGAGCCGAAGCCACTAACACT
β-actin	F	TTCCAGCCATCTTTCTTGGGTA
	R	AGCGTTTACAACCTAACACCA

## Supplementary Information

**Additional file 1:** **Table S1.** Summary of sequencing data.

**Additional file 2:** **Table S2.** Summary of Filtered data.

**Additional file 3:** **Table S3.** Summary of RNASeq Mapping.

**Additional file 4:** **Table S4.** Summary of differentially expressed mRNA in DPV infected DEF cells.

**Additional file 5**: **Table S5.** Summary of differentially expressed lncRNA in DPV infected DEF cells.

**Additional file 6:** **Table S6.** genomic position of lncRNAs.

**Additional file 7:** **Table S7.** Prediction of cis-regulatory targets of lncRNA.

**Additional file 8**: **Table S8.** Prediction of trans-regulatory targets of lncRNA.

**Additional file 9:** **Table S9.** GO enrichment of cis-regulatory targets of DE lncRNA.

**Additional file 10:  Table S10.** KEGG enrichment of cis-regulatory targets of DE lncRNA.

**Additional file 11:** **Table S11**. GO enrichment of trans-regulatory targets of DE lncRNA.

**Additional file 12:** **Table S12.** KEGG enrichment of trans-regulatory targets of DE lncRNA.

**Additional file 13:** **Table S13.** GO enrichment of DE mRNA.

**Additional file 14:** **Table S14.** KEGG enrichment of targets of DE mRNA.

**Additional file 15:** **Table S15.** Prediction of potential targeting viral miRNAs.

**Additional file 16:** **Figure S1.** The predicted targeting regulatory networks of lncRNA-viral miRNA in DPV infected DEFs.

**Additional file 17**: **Figure S2.** Validation of the differential expression of 6 DE lncRNAs by qRT-PCR.

**Additional file 18:** **Figure S3.** Validation of the differential expression of 6 DE mRNAs by qRT-PCR.

## Data Availability

All data generated or analysed during this study are included in this article and its additional files. All RNA-seq data analysed during this study has been deposited in in SRA database (https://www.ncbi.nlm.nih.gov/sra) under accession number: SRR16573633, SRR16573634, SRR16573635, SRR16573636, SRR16573637, SRR16573638.

## Abbreviations

DPV: Duck plague virus
lncRNA: Long non-coding RNA
RNA-seq: RNA-sequencing
CHv: Chinese virulent
DEF: Duck embryonic fibroblast
hpi: Hours post-infection
DE: Differentially expressed
DEG: Differentially expressed genes
MR: Mannose receptor
TLR2: Toll-like receptor 2
KEGG: Kyoto Encyclopedia of Genes and Genomes
GO: Gene Ontology
DP: Duck plague
DEV: Duck viral enteritis
lincRNA: Large intergenic non-coding RNA
RIG-I: Retinoid acid-inducible gene I
TRIM25: Tripartite Motif Containing 25
IFN: Interferon
TDP43: TAR DNA-binding protein 43
IRF3: Interferon regulatory Factor 3
HCV: Hepatitis C virus
DTMUV: Duck Tembusu virus
DMEM: Dulbecco's modified eagle medium
FBS: Fetal bovine serum
PBS: Phosphate buffered saline
MOI: Multiplicity of infection
qRT-PCR: Quantitative Real-time PCR
PRR: Pattern recognition receptors
MF: Molecular function
CC: Cell component
BP: Biological process
MAPK: Mitogen-activated protein kinase
ceRNA: Competing endogenous RNA
MRE: MicroRNA response elements
UL: Unique long
US: Unique short
IRS: Short internal repeat
TRS: Short terminal repeat
HSV: Herpes simplex virus-1
VV: Vaccinia virus
CD2AP: CD2 related protein
PDCoV: Porcine deltacorona virus
HBV: Hepatitis B virus
FMDV: Foot-and-mouth disease virus
BoHV-1: Bovine herpesvirus type 1
KSHV: KS associated herpesvirus
RDUR: RIG-I-dependent IAV upregulated noncoding RNA
IAV: Influenza A virus
GSTM1: Glutathione S-transferase M1
TBK1: TANK binding kinase 1
GOT2: Glutamic acid transaminase 2
PCA: Principal component analysis
TF: Transcription Factor
Pol ll: RNA polymerase II
padj: P-adjusted values
