# Supplementary figures and images for: Differential expression profile and in-silico functional analysis of long noncoding RNA and mRNA in duck embryo fibroblasts infected with duck plague virus
Source: BMC Genomics. 2022 Jul 14;23:509. doi: 10.1186/s12864-022-08739-7 (PMC9281093; doi:10.1186/s12864-022-08739-7)

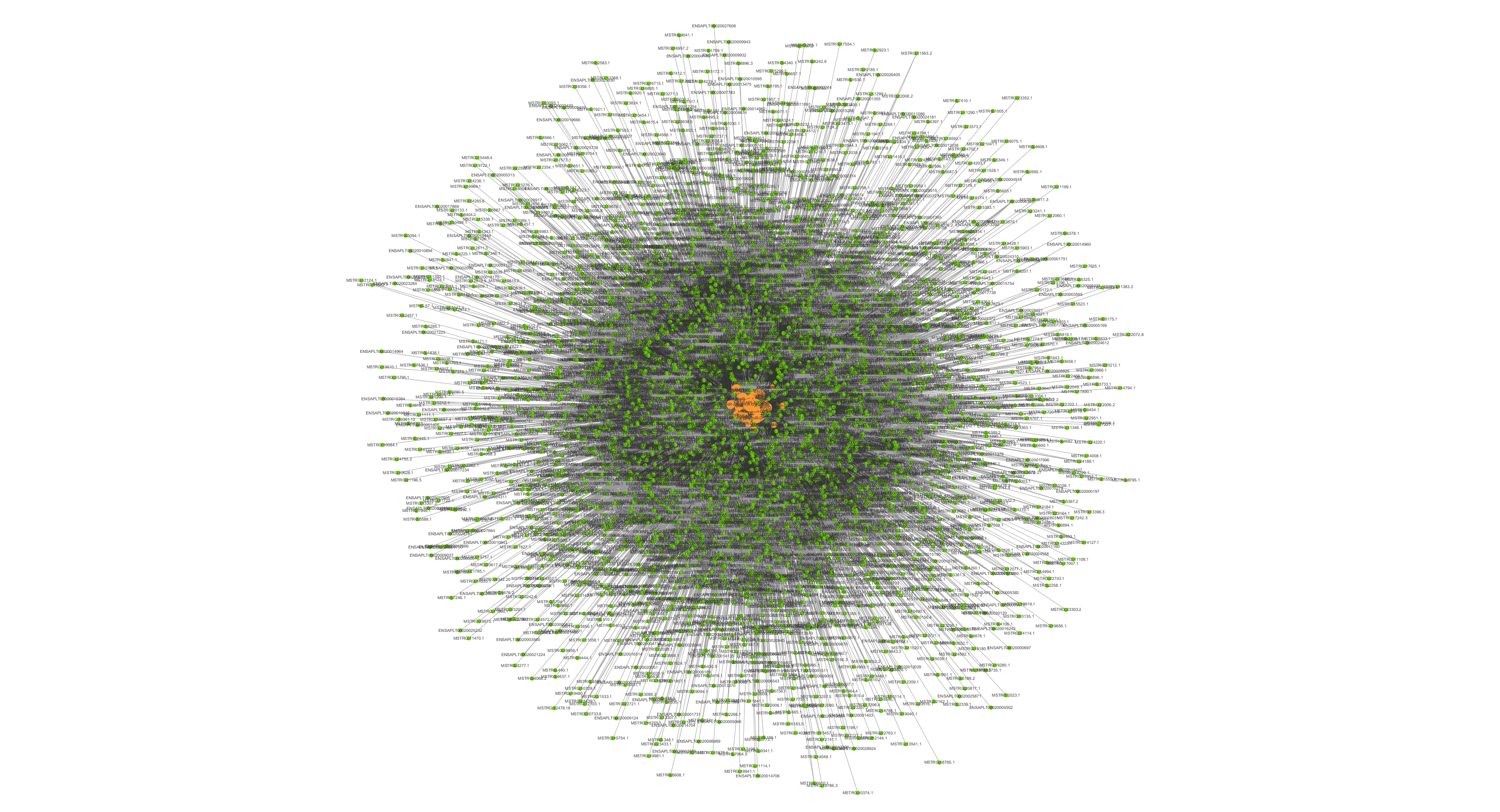

Supplement: Supplementary file 16 — Additional file 16: Figure S1. The predicted targeting regulatory networks of lncRNA-viral miRNA in DPV infected DEFs. [file 12864_2022_8739_MOESM16_ESM.png]

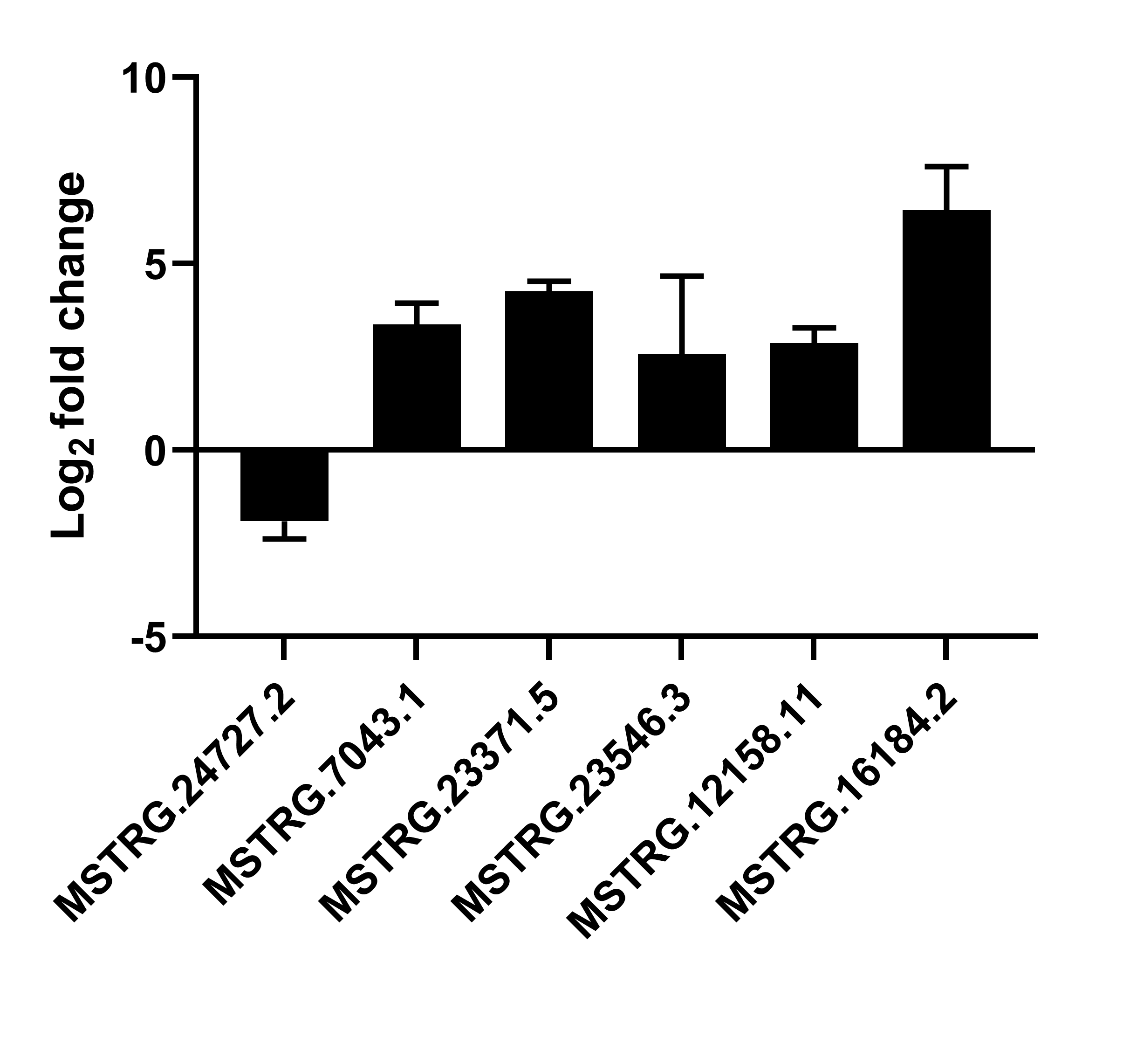

Supplement: Supplementary file 17 — Additional file 17: Figure S2. Validation of the differential expression of 6 DE lncRNAs by qRT-PCR. [file 12864_2022_8739_MOESM17_ESM.png]

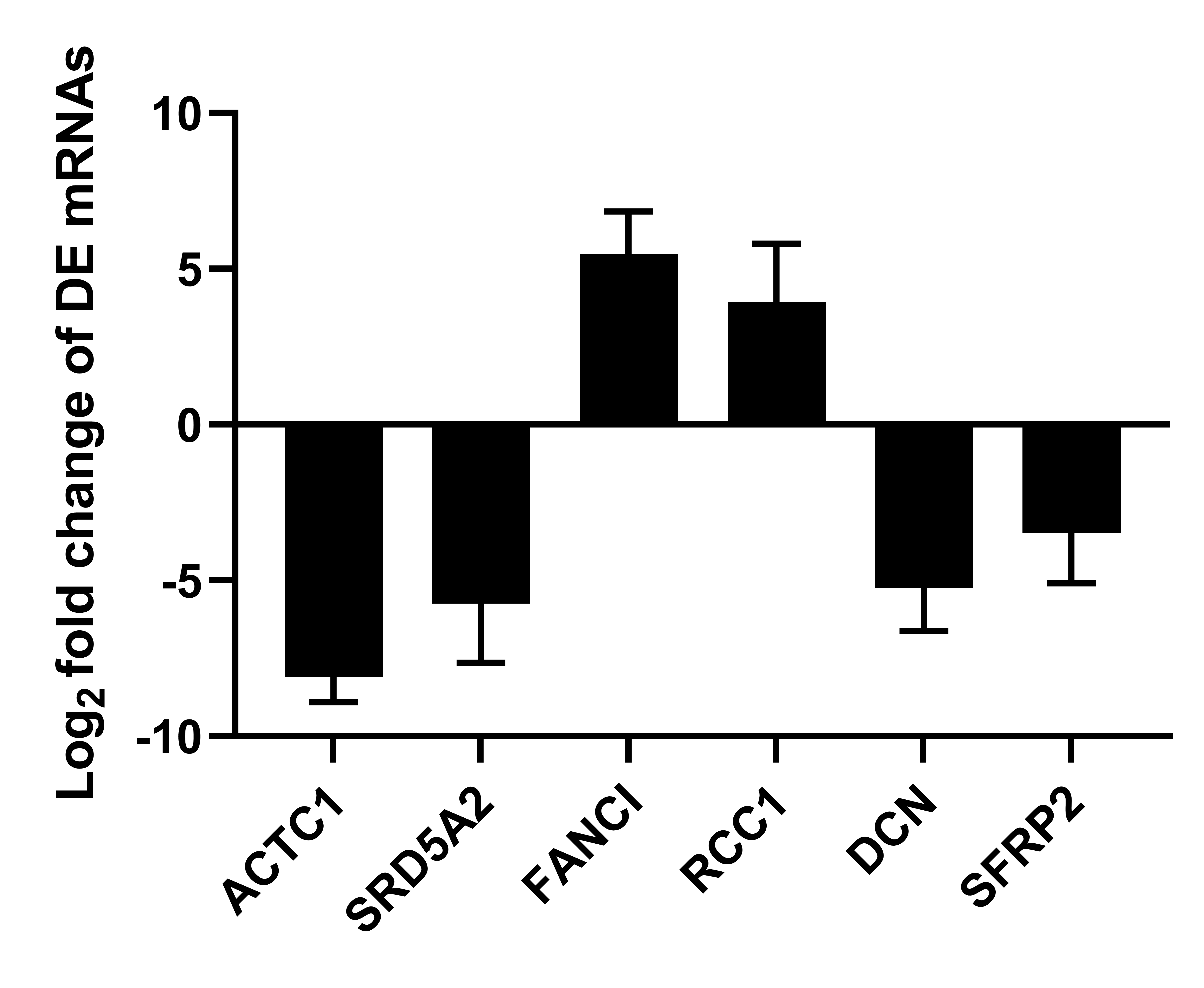

Supplement: Supplementary file 18 — Additional file 18: Figure S3. Validation of the differential expression of 6 DE mRNAs by qRT-PCR. [file 12864_2022_8739_MOESM18_ESM.png]
